# A Call for a Health Data–Informed Workforce Among Clinicians

**DOI:** 10.2196/52290

**Published:** 2024-06-17

**Authors:** Joy Doll, A Jerrod Anzalone, Martina Clarke, Kathryn Cooper, Ann Polich, Jacob Siedlik

**Affiliations:** 1 Department of Mathematics, Creighton University, Omaha, NE, United States; 2 Department of Neurological Sciences, University of Nebraska Medical Center, Omaha, NE, United States; 3 School of Interdisciplinary Informatics, University of Nebraska Omaha, Omaha, NE, United States; 4 Veterans Administration, Phoenix, AZ, United States; 5 Department of Exercise Science and Pre-Health Professions, Creighton University, Omaha, NE, United States

**Keywords:** health data–informed workforce, health data, health informaticist, data literacy, workforce development

## Abstract

A momentous amount of health data has been and is being collected. Across all levels of health care, data are driving decision-making and impacting patient care. A new field of knowledge and role for those in health care is emerging—the need for a health data–informed workforce. In this viewpoint, we describe the approaches needed to build a health data–informed workforce, a new and critical skill for the health care ecosystem.

## Background

Health care has become a data-driven business. It is no longer acceptable that both incoming and current health care professionals and business leaders lack an understanding of the influence data has on health care delivery. The clinician coauthors listed here represent this sphere and are still learning every day. We represent the diverse background of professionals that exist in the health data space, with a wide variety of journeys into this arena [1]. “Health data” is a broad term, often referring to data collected and exchanged in electronic systems. Everyday health data are entered, exchanged, and used to make important decisions from the patient level to the systems level. Health care professionals today need an understanding of the utilization and impact of health data to optimize care delivery and interact with the many systems they encounter daily.

When we entered the health care industry over 20 years ago, we were hopeful clinicians excited to impact patients’ lives. For many of us, we quickly became disillusioned by a system that was driven not by patient outcomes but by reimbursement. Yet, we regained hope with pivotal moments, including when Don Berwick challenged health care organizations to promote quality and evidence-based medicine with the Institute for Healthcare Improvement; the proliferation of electronic health record (EHR) usage leading to the potential to share patient information across systems [2-4]; and the opportunity to move from fee-for-service to value-based payment [5]. We continue to grow in hope, as many openly discuss health equity and social determinants and drivers of health. In addition, the conversations and investments in workforce related to health data knowledge and expertise are ongoing and receiving national attention. Opportunities abound with the expansive growth of artificial intelligence and machine learning.

However, none of these impending innovations can grow and disseminate without understanding data. Gaining an understanding about health data and their use by clinicians is critical to promote the key structural aspects necessary to improve health care delivery, including interoperability, data standards, quality measures, and reimbursement for health outcomes. When we started in health care, the understanding of the impact of health data did not truly and widely exist. In our experience, we find that many clinicians are unconsciously incompetent—lacking a basic understanding of how health data are used, what health data consist of, and where data flow [6]. Unconscious incompetence occurs when the decision makers lack the true information and expertise needed to make an informed decision [6]. This lack of competence causes uninformed decision-making in the health care ecosystem, which causes more challenge. Technology and data become a burden and not a solution.

In health care, a health data–informed workforce is needed to remedy the gaps and make the important connections for positive change. In our experiences and those of our peers, we interact with clinicians who learned about informatics and health data on the job [1]. Many stories start with an interest in data and technology or some savviness with technology. These are individuals willing to lean into innovation and learn through failure. Yet, their learning curve is steep and lacks the efficiency that a health data–informed workforce could address. The understanding of health data has become a shared team value critical to growing and expanding the evidence to support interprofessional practice. Now is the time to move beyond the early adopters and explore how we can expand the health data–informed workforce. We acknowledge previous authors that have called for this momentum to grow and call for ongoing and widespread engagement. In this viewpoint, we attempt to define the health data–informed workforce at the micro-, meso-, and macrolevels. We then offer suggestions for clinicians wanting to level up their competence in health data.

## What Is a Health Data–Informed Workforce?

A health data–informed workforce includes clinicians with a basic understanding of data along with their exchange and influence on decision-making. The ideal would be to move clinicians from being unconsciously incompetent to consciously competent. However, the amount of knowledge expected is overwhelming. The complexity of health data has evolved into the field of health informatics. Multiple studies have indicated that the field of health informatics is diverse, with a wide variety of education and workplace requirements [1,7,8]. Health informatics is a field that explores the use of health data for “scientific inquiry, problem-solving, decision making” with the intent to improve health care delivery and impact [9]. Yet, health data impact every level of health care, from the micro- to macrolevel, calling upon all clinicians to hold a basic understanding.

For the purposes of this viewpoint, we consider the microlevel to be interactions with patients and clinicians; the mesolevel focuses on the infrastructure and systems in place for health data sharing; and the macrolevel addresses the impact of policy on health data. Clinicians who are data informed at each of these levels will improve the impact of health data utility and ensure that decisions made around health data and technology will facilitate positive change.

At the clinician and patient level (ie, the microlevel), data are used to make clinical decisions. The widespread adoption of EHR systems supported by the 21st Century Cures Act and provisions against information blocking in the Office of the National Coordinator of Health Information Technology’s Final Rule place a premium on data, and data literacy, in health care delivery [10]. The availability of data and the ability for patients to access their health data through patient portals and other digital applications can advance shared decision-making, promoting improved health outcomes while empowering patient’s involvement in their care. Yet, EHRs have introduced burden, and many clinicians are under information overload, which can result in health care errors [9,11]. A recent piece in the *Journal of the American Medical Association* titled “Death by Patient Portal” illustrates the love-hate relationship that occurs with much of health technology and data [12]. Data are flowing and being shared, but questions remain on how much and how to make information usable for patients and clinicians. Despite these challenges, data and technology are reported to only continue to grow in health care. This calls on clinicians to know how to access patient data in their EHRs, understand where patients track and record data, and feel comfortable translating health information to multiple levels of digital and health literacy. A health data–informed clinician knows to use tools such as health information exchanges (HIEs) to ensure that they are making clinical decisions with comprehensive patient data beyond the EHR [13]. An HIE extracts data from multiple EHRs and matches that data into a comprehensive patient record. In some health care organizations, HIEs are integrated into the EHR. They can provide quick and comprehensive patient data for clinical decision-making [14]. Due to HIEs, health care becomes more proactive and less reactive when clinicians are aware of a recent emergency department visit, for example. At the same time, HIEs can lead to information overload for providers. In addition, clinicians improve their patient experience when they have information about the patient journey and history, which an HIE can provide [15]. Patients also report a better patient experience when they are not forced to “repeat their story” or re-enter information they have already reported.

Many health care organizations use data for various reasons, including use by health care delivery systems, payers, and academic researchers. When it comes to the mesolevel, the health data–informed workforce needs to understand data governance, including understanding how, why, and when data are shared and recognizing the importance of privacy and security. Patient consent remains important to ensure patients know when and where their data are being shared and how they are being used. In addition, health technology selection and vetting, along with vendor management, is critical. Vendors can offer solutions, yet at the same time, these tools can have unintended consequences from data being entered into multiple systems, causing burden on clinicians and a lack of data completeness in a patient’s record. Clinicians need to recognize the importance of interoperability as it impacts data access and use between systems. Interoperability refers to the ability to exchange data in a useful manner. An interoperable approach reduces double documentation and siloed health data [16]. At the same time, health data have extensive protections under the Health Insurance Portability and Accountability Act (HIPAA), which requires thoughtfulness to the exchange and use of data across systems. We have witnessed too many clinicians enamored with a piece of technology without vetting its ability to further health care. Great technology that further siloes data into multiple systems and lacks expanded adoption can cause more burden and potential patient harm. We need a workforce that questions the benefits and challenges how additional technology and data can actually improve health care delivery along with their interoperability.

Health data–informed clinicians also recognize the importance and value of data standards [17]. Data standards provide a critical foundation for data exchange. Decisions are being made daily in health care organizations without the recognition or use of data standards. One example is choosing to create a health-related social need screener without considerations of existing tools or data standards work, such as that led by the Gravity Project [18]. These approaches further denigrate the system and cause a myriad of challenges to interoperability.

For the macrolevel, understanding and advocating for local and federal policies that support the proliferation of growing workforce expertise is critical for the health data–informed clinician. Clinicians need a basic recognition and understanding of how policy drives health data utility [19]. The gaps in the workforce around health informatics have been identified and acknowledged [7,20,21]. Efforts in this area have been made by the American Medical Informatics Association’s 10×10 program and the federal funding to support the Public Health Informatics and Technology Workforce Program by the Office of National Coordinator of Health Information Technology. These policies and investments provide opportunities to support both current professionals and those entering the workforce, representing examples of the impact of policy on health data.

## How Do Clinicians Level Up?

### Overview

If this sparks something inside you, the next step is to be curious about how to develop into a health data–informed clinician. All clinicians should be on a journey as lifelong learners. Health and health care constantly change, not to mention technology and data use. Becoming more data informed does not mean getting a new degree, even though that is an option. In this next section, we share some basic aspects for those desiring to become a health data–informed clinician. Certainly, we cannot go into extensive depth, but we hope this plants seeds to grow the health data–informed workforce. Some strategies to level up are as follows.

### Get to Know a Health Informaticist

No one can or is expected to know everything, which is why health care is a team sport. One strategy to help build a health data–informed workforce is for clinicians to learn the role of health informaticists. Health informatics “is the interprofessional field that studies and pursues the effective uses of biomedical data, information, and knowledge for scientific inquiry, problem-solving, decision making, motivated by efforts to improve human health” [9]. In other words, health informatics is a wide field focused on health data and their utility to impact health care outcomes. Health informaticists hold expertise in data management, security, privacy, and governance requirements to support safe handling of protected health information [1]. They are also challenged to ensure that health data are interpreted and presented meaningfully to stakeholders, including clinicians; health care leaders; and most importantly, patients [22]. Health informaticists go by different names in different organizations, including clinical informaticist, data analyst, business analyst, etc [23,24]. Their roles and demands may vary based on where they work. However, many organizations have informatics expertise in their organization. The next step would be to include an informaticist as part of the team. They can be invaluable in selecting health technology, vendor management, training and implementation, and project implementation, not to mention data handling! They offer a wide variety of skills to a team interacting with health technology and data, including data extraction, quality metrics, data analysis, dashboard builds, etc [1].

### Use an HIE

HIEs are state-based or regional infrastructures that match data across multiple EHRs to provide a comprehensive patient record [25]. The sophistication of HIEs vary, yet they are a tool available to clinicians in multiple health systems that often go underutilized. In some cases, HIEs can be queried for information on the patient. They can also be used to send and receive information on a patient to allow for more comprehensive decision-making [14,15]. Clinicians can find out if their health care organization is part of their local HIE to gain access and training on how to use an HIE to improve clinical decision-making.

### Recognize the Importance of Data Standards

Clinicians have an important role in entering health data, which impacts the ability to analyze data from health data utilities. Recognizing the importance of the use of appropriate data standards is important for clinicians. In the informatics field, you often hear the term “garbage in, garbage out,” and much effort has been made to extract and clean data to show the impact of quality payment programs, which have induced new health care system burden. Clinicians can work with their informatics team to ensure documentation is structured in a meaningful way. The United States Core Data for Interoperability offers guidance around common ways to document that can promote data sharing [26].

### Get Some Training

There is a variety of professional organizations that can support the learning and growing of professionals targeted at clinicians. These organizations offer conferences, web-based trainings, and certifications. Some federal resources also exist, including the Office of the National Coordinator for Health Information Technology, who offers webinars and other valuable resources. Table 1 calls out some of these resources. Each organization offers a variety of training opportunities. Another option is to seek a mentor in health informatics, partnering with someone with experience to learn from.

**Table 1.** Organizations and resources.

Resource	Website
American Health Information Management Association (AHIMA)	[27]
American Medical Informatics Association (AMIA)	[28]
Civitas Networks for Health	[29]
Healthcare Information and Management Systems Society (HIMSS)	[30]
Office of the National Coordinator for Health Information Technology	[31]

For some, upskilling may be entering the field of health informatics. Academic programs exist in health information management and health informatics across the country. Many professional organizations offer discipline-tailored programming in health informatics specifically in medicine and nursing. Many programs offer web-based options and teach core skills.

It is normal to feel intimidated by the terminology and concepts. However, it is important to remember that health data are being used to drive lots of decisions. Garnering a basic understanding will improve clinical skills and help with patient advocacy to improve care delivery. Everyone can take some simple steps to become more health data informed.

## What Can Educators Do?

### Overview

It is impossible to know everything about the field of informatics and health data. Instead, the intent should not be about teaching all the skills but instead the critical thinking skills necessary to consider how and why technology and data can be used in health care. As a society, we need to cultivate minds that can think and problem solve for a future we do not yet exist in. As educators, we need to encourage the ability to embrace ambiguity and innovation while recognizing that human beings approach these elements in different ways that can cultivate adoption at different rates of speed. Educating a health data–informed workforce requires educators to recognize that technical and technology skills are important but not enough. The focus should include the following.

### Promotion of Data Literacy

Basic data literacy involves understanding how data can be used to effect positive change in patient outcomes, cost reduction, and mitigation of caregiver burnout, among other applications. Data literacy is the ability to read and understand data. For those advanced in this area, data literacy includes communicating and sharing data in ways appropriate to the audience. Health data literacy in an informed health care workforce includes training on effective data management throughout the health data life cycle and how to traverse the knowledge discovery process, from data to information to knowledge and, ultimately, wisdom and actionable insights. The Data, Information, Knowledge and Wisdom Model provides a theoretical framework that spans from reviewing data to applying data in impactful ways [32]. A significant amount of health data is collected, and deciding what to do with it requires a deeper understanding. Educators should push learners to move beyond reviewing data to deeply engaging with them in meaningful ways to improve health care.

### Ethical Use of Health Data

Data, especially health data, require a high level of care and stewardship. Educators need to focus on the ethics of data use; data governance, including privacy and security along with appropriate data-sharing strategies; and the importance of recognizing data literacy for key stakeholders, including patients, policy makers, payers, clinicians, and health care executives. Data brokering and its impact on health care continue to evolve.

The infusion of artificial intelligence will continue to generate new ethical questions, opportunities, and concerns [33]. In addition, gaps in data and new data areas such as social determinants and drivers of health offer new and interesting challenges to consider [18]. Furthermore, innovation should always be grounded in asking the “what if” questions to ensure that ethical considerations are always an aspect of data use.

### Focus on Data Utility

Health data are being collected at a momentous rate. Educators must focus on preparing a health data–informed workforce to recognize the utility of data for the audience. This must also be considered in implementing health information technology mechanisms focused on user experience and human-centered design to ensure that health data are used thoughtfully and ethically. Data standards are also critical to utility, such as the United States Core Data for Interoperability [26]. We have witnessed many implementations without the consideration of data standards, causing barriers to interoperability that can produce harm in patient care. We can name multiple examples where technology is purchased without even considering how systems will share or integrate data, causing myriad other challenges in health care.

### Recognize the Impact of the System

Health care is a large system within systems. Health technology and data are driven by systems, whether they be legal, policy, or reimbursement. Implementing data and technology without a strong understanding of the mechanisms and systems thinking is problematic. A health data–informed workforce recognizes the many layered systems impacting health information technology and data use implementation. Ensuring that the workforce engages in systems thinking and searching for “the why” in implementation and data use is a critical skill. In addition, clinicians should not feel disempowered and instead recognize the role they can play at the microlevel in patient interactions to improve the use of health data for improved outcomes.

## Conclusion

Our hope is to promote a conversation and spark innovation around the need to expand and grow the health data–informed workforce. We certainly cannot provide every piece of advice or suggestion here. Yet, we hope to spark a revolution to grow the cadre of passionate advocates for the proliferation of health data and technology in ways that truly support equity, reduce burden, and improve health care delivery. Additionally, we are not saying that data skills are not critical—they are. We recognize that we need more than that. We need a workforce that asks questions about where data go and how they are used and that becomes more informed on the data tools of their patients. We need a health data–informed workforce now and into the future.

## Abbreviations

EHR: electronic health record
HIE: health information exchange
HIPAA: Health Insurance Portability and Accountability Act
